# d-Amphetamine Transdermal System in Treatment of Children and Adolescents with Attention-Deficit/Hyperactivity Disorder: Secondary Endpoint Results and *Post Hoc* Effect Size Analyses from a Pivotal Trial

**DOI:** 10.1089/cap.2023.0005

**Published:** 2023-06-16

**Authors:** Andrew J. Cutler, Katsumi Suzuki, Brittney Starling, Kanan Balakrishnan, Marina Komaroff, Suzanne Meeves, Mariacristina Castelli, Ann Childress

**Affiliations:** ^1^ Department of Psychiatry, SUNY Upstate Medical University, Neuroscience Education Institute, Lakewood Ranch, Florida, USA.; ^2^ Product Development, Noven Pharmaceuticals, Inc., Jersey City, New Jersey, USA.; ^3^ Center for Psychiatry and Behavioral Medicine, Inc., Las Vegas, Nevada, USA.

**Keywords:** ADHD, amphetamine, transdermal, effect size, number needed to treat

## Abstract

**Objectives:**

Amphetamines are a preferred treatment for attention-deficit/hyperactivity disorder (ADHD), with the dextroamphetamine transdermal system (d-ATS) providing an alternative to oral formulations. A pivotal trial of d-ATS in children and adolescents with ADHD met primary and key secondary endpoints. This analysis reports additional endpoints and safety findings from the pivotal trial and evaluates effect size and number needed to treat (NNT) for d-ATS.

**Methods:**

In this study, a 5-week, open-label dose-optimization period (DOP) preceded a 2-week, randomized, crossover double-blind treatment period (DBP). Eligible patients received d-ATS 5 mg during the DOP, with weekly evaluations for increase to 10, 15, and 20 mg (equivalent to labeled doses of 4.5, 9, 13.5, and 18 mg/9 hours, respectively) until reaching and maintaining the optimal dose, which was utilized for the DBP. Secondary endpoints included assessment of Attention-Deficit/Hyperactivity Disorder Rating Scale IV (ADHD-RS-IV), Conners' Parent Rating Scale Revised Short Form (CPRS-R:S), and Clinical Global Impression (CGI) scores. NNT was calculated for ADHD-RS-IV and CGI-Improvement (CGI-I). Safety assessments included treatment-emergent adverse events (TEAEs) and dermal safety.

**Results:**

In total, 110 patients entered the DOP, with 106 patients randomized (DBP). During the DBP, the least-squares mean (95% confidence interval) difference for d-ATS versus placebo in ADHD-RS-IV total score was −13.1 (−16.2 to −10.0; *p* < 0.001), with effect size of 1.1 and NNT of 3 for ADHD-RS-IV remission, ≥30% improvement, and ≥50% improvement. Significant differences between placebo and d-ATS were also observed for CPRS-R:S and CGI-I scales (*p* < 0.001), with NNT of 2 for CGI-I response. Most TEAEs were mild or moderate, with three leading to study discontinuation in the DOP and none in the DBP. No patients discontinued due to dermal reactions.

**Conclusions:**

d-ATS was effective in treating ADHD in children and adolescents, meeting all secondary endpoints, with a large effect size and NNT of 2–3 to achieve a clinically meaningful response. d-ATS was safe and well tolerated, with minimal dermal reactions.

**Clinical Trial Registration:**

NCT01711021.

## Introduction

Attention-deficit/hyperactivity disorder (ADHD) is a chronic neurodevelopmental disorder characterized by persistent inattention and/or hyperactivity/impulsivity that interferes with function or development and negatively impacts social, academic, and occupational activities (American Psychiatric Association, 2013; Bélanger et al., 2018). ADHD is estimated to occur in ∼7% of children and adolescents worldwide (Polanczyk et al., 2007; Thomas et al., 2015). In the United States, ∼60% of children and adolescents with ADHD are receiving approved medications; however, treatment adherence and persistence remain low, signaling unmet needs in the treatment of children and adolescents with ADHD (Danielson et al., 2018; Gajria et al., 2014; Schein et al., 2022).

Stimulants such as methylphenidate and amphetamine are recognized as the most commonly prescribed and effective treatments for ADHD in children and adolescents (Cortese et al., 2018; Huss et al., 2017; Pliszka, 2007; Wolraich et al., 2019). Although methylphenidate and amphetamine have proved highly effective in relieving ADHD, ∼40% of patients preferentially respond to or tolerate only one of these drugs, highlighting a need to provide additional treatment options, including multiple formulations, for treating ADHD (Wolraich et al., 2019).

Transdermal formulations offer a number of features that patients and caregivers may view as advantages over oral formulations, including easier treatment of individuals who have difficulty swallowing oral medications, potential for fewer gastrointestinal side effects, flexibility in treatment duration by varying patch wear times, and visual confirmation of adherence to treatment (Findling and Dinh, 2014). A transdermal formulation of methylphenidate has been approved for the treatment of ADHD in the United States for several years (Daytrana Prescribing Information, 2022). However, in crossover studies, as many as 25% of patients with ADHD have been shown to respond better to amphetamines than methylphenidate, with similar results observed in some head-to-head comparisons (Arnold, 2000; Newcorn et al., 2017; Soutullo et al., 2013; Stein et al., 2011). Therefore, an unmet need for a transdermal amphetamine formulation remains.

The dextroamphetamine transdermal system (d-ATS) was developed as an alternative to oral amphetamine formulations and is the first and only transdermal amphetamine patch approved by the Food and Drug Administration (FDA) for use in adults and pediatric patients (children and adolescents) 6 years and older (Noven Therapeutics, 2022).

A pivotal randomized controlled trial of d-ATS in children and adolescents with ADHD was conducted, and its primary endpoint (efficacy of d-ATS compared with placebo, as measured by the Swanson, Kotkin, Agler, M-Flynn, and Pelham scale [SKAMP] total score) and key secondary endpoints were met (Cutler et al., 2022). In this study, d-ATS treatment resulted in a significant improvement in SKAMP total score, with an overall least-squares (LS) mean difference (95% confidence interval [CI]) versus placebo of −5.87 (6.76 to −4.97; *p* < 0.001) over 12 hours. Improvements in total SKAMP score were observed from 2 through 12 hours after application (*p* ≤ 0.003 for all). Significant improvements in Permanent Product Measure of Performance (PERMP)-A and PERMP-C scores were also observed from 2 to 12 hours after d-ATS treatment compared with placebo (*p* < 0.001 for all). d-ATS was observed to be safe and well tolerated, with a systemic safety profile similar to that observed with oral amphetamines and no application-site reactions leading to discontinuation (Cutler et al., 2022).

When considering treatment options for ADHD, clinicians must weigh the potential benefit of a given treatment, which is generally indicated by response, against its potential risks, such as safety and/or tolerability issues. Measures such as effect size and number needed to treat (NNT) can be used to quantify these potential benefits by providing clinical meaningfulness to changes in rating scale scores. Effect sizes estimate the efficacy of an active intervention to quantify and communicate the clinical relevance of statistically significant trial results. Cohen's effect size for comparison between two groups (active vs. placebo) is classified as small (0.20), medium (0.50), or large (0.80) (Cohen, 1992). NNT describes how many patients would need to be treated with one intervention instead of a comparator (placebo in this instance) to achieve one additional positive outcome of interest (Citrome and Ketter, 2013).

To further assess the efficacy and safety of d-ATS in the treatment of 6–17-year-old children and adolescents with ADHD in a randomized laboratory classroom study, we report additional secondary endpoints from the pivotal study, including Attention Deficit/Hyperactivity Disorder Rating Scale IV (ADHD-RS-IV), Conners' Parent Rating Scale Revised Short Form (CPRS-R:S), and Clinical Global Impression-Improvement (CGI-I) and -Severity (CGI-S) scores. In a *post hoc* analysis, the ADHD-RS-IV endpoint was also used to evaluate effect size, and the ADHD-RS-IV and CGI-I endpoints were used to evaluate NNT for d-ATS.

## Methods

### Patients and study design

The study described herein was approved by the Western Institutional Review Board and the UC Irvine Institutional Review Board. This study included a 5-week, open-label dose-optimization period (DOP) followed by a 2-week, randomized, crossover double-blind treatment period (DBP), with details reported previously (Cutler et al., 2022). Briefly, patients were children and adolescents 6–17 years of age with a primary diagnosis of ADHD combined, hyperactive/impulsive subtype, or predominately inattentive subtype. At screening and baseline, patients' ADHD-RS-IV scores must have been ≥90% of the general population of children by age and gender. Patients currently taking ADHD medication adequately providing symptom control, as well as those known to not respond to or tolerate amphetamine treatment, were excluded.

After a 3-day washout period, all eligible patients entered the 5-week DOP, which evaluated 4 doses of d-ATS: 5, 10,15, and, 20 mg (equivalent to approved doses of 4.5 mg/9 hours, 9 mg/9 hours, 13.5 mg/9 hours, and 18 mg/9 hours, respectively) (Xelstrym Prescribing Information, 2022). Doses were applied to the hip for 9 hours per day on alternating sides. All patients initiated daily applications of d-ATS at a dose of 5 mg. Patients were evaluated weekly (Visits 1–5) during the DOP for possible dose adjustment by one dose level (e.g., from 5 to 10 mg) until an optimal dose was reached. Once reached, the optimal dose was maintained for the DOP. During the 2-week DBP, patients reaching the optimal dose were randomized to receive double-blind treatment or placebo on Visit 6 and then crossed over to receive the other study treatment for Visit 7.

### Study assessments

Secondary objectives for this study included assessment of efficacy through the ADHD-RS-IV scale, CPRS-R:S scale, and CGI-I and CGI-S scores. Analysis of these secondary assessments was performed on the Full Analysis Set, which included all randomized patients who received at least one dose of study medication.

Safety outcomes, including treatment-emergent adverse events (TEAEs) and dermal safety, were based on the Safety Population, which included all subjects who took at least one dose of study medication and had at least one postdose safety measurement (including dermal assessments).

### Statistical analysis

LS means, 95% CIs, and *p*-values were calculated from a linear mixed model that included ADHD-RS-IV or CPRS-R:S scores for each time point as dependent variables, sequence, and treatment as fixed effects, and subject as the random effect.

Model-based LS mean effect sizes were assessed for ADHD-RS-IV total score and the inattention and hyperactivity–impulsivity subscale scores *post hoc*. Analyses were performed on the full study population and subgroups of children (6–12 years of age) and adolescents (13–17 years of age). Effect size was defined as the difference in LS mean score between treatment arms divided by the square root of the mean square error obtained from the model (Curtin et al., 2002). The McNemar test for paired samples was used for ADHD-RS-IV and CGI-I responder analysis. Remission has been defined as an ADHD-RS-IV total score ≤18 and is the cut point used in this *post hoc* analysis (Mattingly et al., 2013; Weiss et al., 2019). Responders were defined as patients with ADHD-RS-IV total score reductions from baseline ≥30% and ≥50% (Dittmann et al., 2014; Mattingly et al., 2013; Weiss et al., 2019; Weiss et al., 2018). CGI-I responders were defined as patients who were assessed by investigators as “very much improved” or “much improved.”

NNT was calculated *post hoc* using the inverse of the difference in proportions between treatment groups for ADHD-RS-IV and CGI-I responders.

## Results

### Baseline characteristics

In total, 110 subjects were enrolled in the DOP, with optimized doses achieved for 107 patients. The *n* (%) of patients optimized to each d-ATS dose was 7 (7) for 5 mg, 35 (33) for 10 mg, 42 (39) for 15 mg, and 23 (21) for 20 mg (Cutler et al., 2022). Four patients did not complete the DOP, although no patients discontinued from the DOP because of lack of efficacy. One patient withdrew consent following the DOP; therefore, 106 patients were randomized in the DBP.

Details of the demographics and baseline characteristics for the population have been reported in detail previously. Briefly, 69% of patients were male, and weight, height, and body mass index were balanced across treatment sequence groups. Mean (standard deviation [SD]) baseline ADHD-RS-IV total score for the Safety Population (all patients receiving at least one dose of study medication and having at least one postdose measurement) was 38.3 (8.6), assessed before the start of the DOP (Cutler et al., 2022).

### Efficacy

**FIG. 1.**
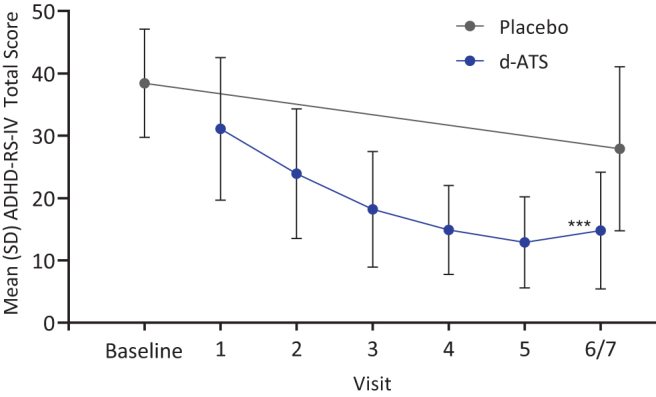
ADHD-RS-IV total scores by visit during the DOP and DBP (Full Analysis Set). DOP comprised Visits 1–5, and DBP comprised Visit 6/7. ***p < 0.001, based on LS means using a linear mixed model, including ADHD-RS-IV for each visit and sequence and treatment as fixed effects. ADHD-RS-IV, Attention-Deficit/Hyperactivity Disorder Rating Scale IV; DBP, double-blind treatment period; DOP, dose-optimization period; LS, least-squares; SD, standard deviation.

Throughout the DOP, mean ADHD-RS-IV total scores progressively improved from baseline, with mean (SD) changes from baseline of −7.4 (8.6) at Visit 1, −14.5 (9.0) at Visit 2, −20.3 (9.4) at Visit 3, −23.6 (9.3) at Visit 4, and −25.6 (9.2) at Visit 5. For both classroom days during the DBP (Visits 6 and 7 combined), ADHD-RS-IV total scores showed improvement from baseline after treatment with d-ATS compared with placebo, with a mean (SD) change from baseline of −23.4 (11.1) with d-ATS and −10.4 (11.1) with placebo. A significant difference (d-ATS vs. placebo) in LS mean (95% CI) change from baseline in ADHD-RS-IV total score was observed: −13.1 (−15.9 to −10.2; *p* < 0.001). ADHD-RS-IV total scores from baseline through Visit 6/7 are shown in Figure 1. Similarly consistent improvements from baseline in ADHD-RS-IV inattention and hyperactivity–impulsivity subscale scores were observed during the DOP.

During the DBP, significant differences (d-ATS vs. placebo) in LS means of ADHD-RS-IV total scores and the inattention and hyperactivity–impulsivity subscales were observed for the full study population and subgroups of children and adolescents (Table 1). Effect size, analyzed *post hoc*, for ADHD-RS-IV total score in the full study population was observed to be 1.1, with effect sizes of 1.2 and 0.9 in the inattention and hyperactivity–impulsivity subscales, respectively (Table 1). Effect sizes on ADHD-RS-IV total score and the inattention subscale observed in children and in adolescents were similar to those observed in the full population. For the ADHD hyperactivity–impulsivity subscale, the effect size observed *post hoc* was slightly higher for children (1.0) than for adolescents (0.7), who started with a lower baseline score on this subscale (Table 1). d-ATS had an NNT of 3 for remission, ≥30% improvement, and ≥50% improvement (clinically meaningful change) as measured by ADHD-RS-IV score (Table 2).

**Table 1.** Least-Squares Mean (Standard Error) Attention-Deficit/Hyperactivity Disorder Rating Scale IV Total and Subscale Scores: Difference and Model-Based Effect Size in the Double-Blind Treatment Period

	Baseline Mean (SD)	d-ATS LS mean (SE)*^a^ n* = 106	Placebo LS mean (SE)*^a^ n* = 106	Difference (d-ATS–Placebo) in LS mean (95% CI)*^a^*	Model-based effect size*^b^*
Children and adolescents (*n* = 106)					
ADHD-RS-IV total score	38.4 (8.7)	14.8 (1.1)	27.9 (1.1)	−13.1 (−16.2 to −10.0)^***^	1.1
ADHD inattention subscale	22.7 (3.4)	9.0 (0.6)	16.3 (0.6)	−7.3 (−8.9 to −5.6)^***^	1.2
ADHD hyperactivity–impulsivity subscale	15.7 (7.2)	5.8 (0.7)	11.6 (0.7)	−5.8 (−7.6 to −4.0)^***^	0.9
Children (*n* = 76)					
ADHD-RS-IV total score	39.7 (7.3)	15.9 (1.4)	30.0 (1.4)	−14.1 (−17.9 to −10.4)^***^	1.2
ADHD inattention subscale	22.7 (3.2)	9.2 (0.7)	16.8 (0.7)	−7.6 (−9.6 to −5.6)^***^	1.2
ADHD hyperactivity–impulsivity subscale	17.1 (6.2)	6.8 (0.8)	13.3 (0.8)	−6.5 (−8.6 to −4.4)^***^	1.0
Adolescents (*n* = 30)					
ADHD-RS-IV total score	35.1 (10.9)	12.1 (1.9)	22.6 (1.8)	−10.5 (−15.8 to −5.3)^***^	1.1
ADHD inattention subscale	22.8 (3.8)	8.6 (1.0)	15.2 (1.0)	−6.6 (−9.5 to −3.7)^***^	1.2
ADHD hyperactivity–impulsivity subscale	12.3 (8.5)	3.5 (1.1)	7.4 (1.1)	−4.0 (−7.2 to −0.8)^*^	0.7

^*^ *p* < 0.05; ^**^p < 0.01; ^***^p < 0.001.

^a^ LS means, 95% CIs and *p*-values were obtained from a linear mixed model that included the ADHD-RS-IV total scores for each time point, sequence, and treatment as fixed effects, and subject as the random effect using a variance components correlation structure.

^b^ Effect sizes based on the change in ADHD-RS-IV scores from baseline were calculated as the difference in LS mean score between treatment arms divided by square root of the mean square error obtained from the model.

ADHD-RS-IV, Attention-Deficit/Hyperactivity Disorder Rating Scale-IV; CI, confidence interval; d-ATS, dextroamphetamine transdermal system; DBP, double-blind period; LS, least-squares; SD, standard deviation; SE, standard error.

**Table 2.** Attention-Deficit/Hyperactivity Disorder Rating Scale-IV Responders in the Double-Blind Treatment Period

	d-ATS (*n* = 104)*^a^ n *(%)	Placebo (*n* = 104)*^a^ n *(%)	*p*	NNT*^b^*
ADHD-RS-IV remission responders (≤18 at endpoint)	73 (70)	24 (23)	<0.0001	3
ADHD-RS-IV ≥30% reduction from baseline responders	93 (89)	44 (42)	<0.0001	3
ADHD-RS-IV ≥50% reduction from baseline responders	71 (68)	25 (24)	<0.0001	3

^***^ *p* < 0.0001 for d-ATS versus placebo, based on the McNemar test for paired samples.

^a^ One hundred and six patients were randomized in the DBP; 104 patients had assessments from both treatment periods.

^b^ Inverse of difference in proportions between treatment groups.

ADHD-RS-IV, Attention-Deficit/Hyperactivity Disorder Rating Scale-IV; d-ATS, dextroamphetamine transdermal system; DBP, double-blind period; NNT, number needed to treat.

**FIG. 2.**
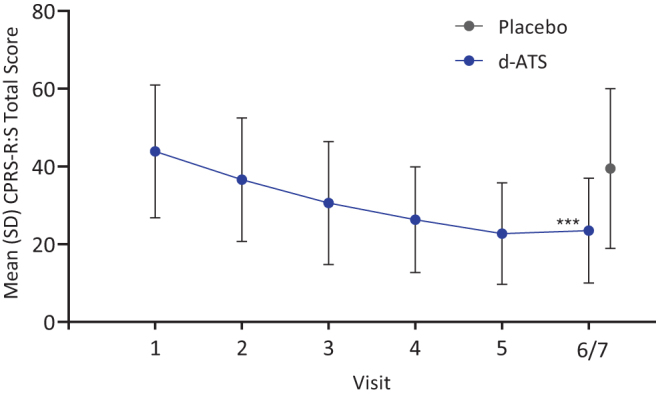
CPRS-R:S total scores by visit during the DOP and DBP (Full Analysis Set). DOP comprised Visits 1–5, and DBP comprised Visit 6/7. ***p < 0.001, based on a linear mixed model, including CPRS-R:S for each visit and sequence and treatment as fixed effects. CPRS-R:S, Conners' Parent Rating Scale Revised Short Form; DBP, double-blind treatment period; DOP, dose-optimization period; SD, standard deviation.

CPRS-R:S total scores also progressively improved throughout the DOP, with mean (SD) scores of 43.9 (17.1) at Visit 1, 36.6 (15.9) at Visit 2, 30.6 (15.8) at Visit 3, 26.3 (13.6) at Visit 4, and 22.7 (13.1) at Visit 5 (Fig. 2). During the DBP (Visits 6/7), mean (SD) CPRS-R:S total scores were 23.5 (13.5) with d-ATS and 39.5 (20.6) with placebo (LS mean difference [95% CI]: −16.1 [−20.9 to −11.2]; *p* < 0.001) (Fig. 2).

**FIG. 3.**
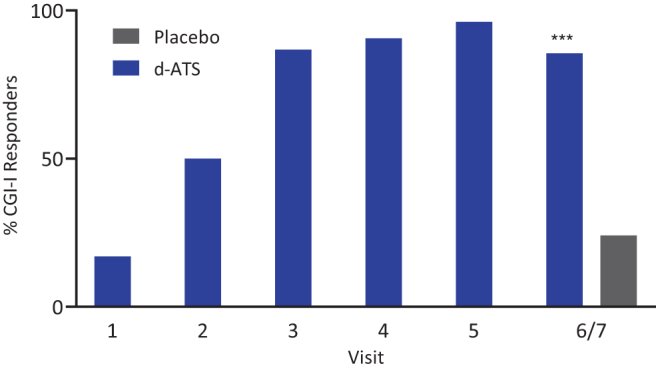
Percentage of CGI-I responders by visit during the DOP and DBP (Full Analysis Set). DOP comprised Visits 1–5, and DBP comprised Visit 6/7. Responder includes the categories “very much improved” and “much improved.” ***p < 0.001, based on the McNemar test for paired samples. CGI-I, Clinical Global Impression-Improvement; DBP, double-blind treatment period; DOP, dose-optimization period.

With respect to CGI scores, according to CGI-S assessments, most patients were moderately ill (33%) or markedly ill (61%) at baseline. During the DOP, the proportion of patients considered CGI-I responders (assessed by investigators as “very much improved” or “much improved”) increased from Visit 1 (17% [18/106]) to Visit 5 (96% [102/106], Fig. 3). During the DBP (Visits 6/7), significantly more patients treated with d-ATS (86% [89/104]) were assessed as CGI-I responders than patients treated with placebo (24% [25/105]; *p* < 0.001) (Fig. 3). d-ATS had an observed (*post hoc*) NNT of 2 for CGI-I response.

### Safety

Safety outcomes have been reported in detail previously (Cutler et al., 2022). Across all doses, 95% (105/110) of patients reported TEAEs during the DOP, although most were mild or moderate, with 3% (3/110) of patients reporting severe TEAEs. Three patients discontinued the study due to TEAEs during the DOP (moderate abdominal pain, irritability, and moderate appetite loss). No patients discontinued due to TEAEs in the DBP. No patients discontinued due to dermal reactions in either phase.

## Discussion

The reported data indicate that d-ATS was effective in the treatment of ADHD in children and adolescents, meeting all secondary endpoints. Throughout the DOP, treatment with d-ATS resulted in consistent improvement as measured by ADHD-RS-IV score, with a significant improvement versus placebo observed during the DBP. A significant difference between placebo and d-ATS treatment was also observed on the CPRS-R:S scale during the DBP, and significantly more patients treated with d-ATS versus placebo were considered responders according to the CGI-I scale. As reported previously, d-ATS met its primary endpoint in a pivotal phase 2 study, resulting in significant improvement versus placebo in ADHD symptoms from 2 through 12 hours postdose, as measured by SKAMP total score (Cutler et al., 2022). Improvements in PERMP scores were also observed from 2 to 12 hours postdose with d-ATS treatment versus placebo (Cutler et al., 2022). This report demonstrates that additional secondary endpoints from the phase 2 pivotal study were met.

d-ATS has been observed to be safe and well tolerated. A systemic safety profile consistent with oral amphetamines was observed, according to this and previous reports (Cutler et al., 2022). Three TEAEs resulted in study discontinuation during the DOP: moderate abdominal pain, irritability, and moderate appetite loss. As previously reported, the most common TEAEs experienced with d-ATS were decreased appetite, insomnia, and headache during both the DOP and DBP (Cutler et al., 2022). No patients discontinued the study due to dermal reactions, and as previously reported, dermal reactions were minimal overall, with meaningful skin irritation occurring in 2 (2%) patients (Cutler et al., 2022).

These efficacy results are also supported by a *post hoc* analysis, further indicating the efficacy of d-ATS in this indication and patient population. Data from the pivotal study suggest that effect sizes of d-ATS on reduction in ADHD-RS-IV total score and the inattention and hyperactivity–impulsivity subscales ranged from 1.2 to 0.7, which would be considered large based on Cohen's classification (Cohen, 1992). Of note, we observed a higher effect size on the hyperactivity–impulsivity subscale among children (1.0) than adolescents (0.7), consistent with clinical observations and findings from longitudinal studies showing that the specific symptoms of hyperactivity and impulsivity tend to diminish as children move into adolescence (Biederman et al., 2000; Francx et al., 2015; Hart et al., 1995; Todd et al., 2008).

The NNT values calculated for the selected endpoints indicate the clinical relevance of d-ATS treatment. Only three patients needed to be treated with d-ATS instead of placebo to observe one additional patient achieving a clinically meaningful change, that is, a 50% or greater reduction from baseline in ADHD-RS-IV score. Only two patients needed to be treated with d-ATS instead of placebo to observe one additional patient experiencing “much improved” or “very much improved” ADHD symptoms, as measured by CGI-I scores.

The observed d-ATS efficacy is comparable to oral preparations of amphetamine and methylphenidate, with an observed effect size and NNT similar to those reported elsewhere. In a study investigating the efficacy of oral lisdexamfetamine and osmotic-release oral system methylphenidate in children and adolescents with ADHD, effect sizes for LS mean change in ADHD-RS-IV total score versus placebo were 1.80 and 1.26, respectively (Soutullo et al., 2013). The NNT to observe improvement was 2 and 3, respectively (Soutullo et al., 2013). Another study investigating the efficacy of oral lisdexamfetamine compared with placebo in adults with ADHD reported effect sizes on ADHD-RS-IV total scores and the hyperactivity–impulsivity and inattention subscales ranging from 1.0 to 1.2 (Wigal et al., 2011). A study of amphetamine extended-release oral suspension in children with ADHD reported effect sizes of 1.8 for SKAMP-Combined score and 1.1 and 1.3 for the attention and deportment subscales, respectively (Childress et al., 2018).

One limitation of this study is its relatively short duration. Furthermore, the classroom setting does not perfectly replicate a typical elementary or secondary classroom, which limits the generalizability of the results. Although a carryover effect was investigated for the primary endpoint (Cutler et al., 2022), it was not addressed for the secondary endpoints, which is another limitation of the analysis. A limitation of the *post hoc* analysis is that number needed to harm (NNH) and likelihood to be helped or harmed (LHH) were not calculated from the pivotal study data. These measures would offer a more complete picture of the clinical utility of d-ATS; however, analysis of NNH and LHH would require a study that begins with a DBP rather than an open-label DOP. In addition, calculating NNH and LHH is complicated by the fact that there were no discontinuations due to TEAEs during the DBP. Lastly, there is no consensus in the literature on what change in ADHD-RS-IV score should be used to define improvement or remission.

This analysis defined ≥30% as the lower threshold for ADHD-RS-IV response; however, some patients achieving a 30% change in ADHD-RS-IV score, particularly those with severe symptoms at baseline, may not experience clinical improvement (i.e., a CGI-I response) (Weiss et al., 2019).

Transdermal delivery has the potential to provide several advantages over oral medications, including avoiding gastrointestinal side effects, allowing flexibility in treatment duration, providing an additional treatment option for patients who may not be able or willing to swallow medications, and enabling a simple visual check for treatment compliance (Findling and Dinh, 2014). A methylphenidate transdermal patch is currently available in the United States, and d-ATS was recently approved by the FDA for treatment of ADHD (Mattingly et al., 2017; Noven Therapeutics, 2022). As the first and only transdermal amphetamine product available in the United States for this indication.

## Conclusion

d-ATS represents an important innovation for the known population of patients with ADHD who respond better to amphetamine than methylphenidate (Arnold, 2000; Newcorn et al., 2017; Soutullo et al., 2013; Stein et al., 2011). Given its observed efficacy and potential advantages over oral formulations, d-ATS may represent a valuable treatment option for children, adolescents, and adults with ADHD.

## Clinical Significance

Methylphenidate and amphetamines have shown high efficacy in treating ADHD in children and adolescents; however, approximately 40% of patients with ADHD respond to only one of these drugs, indicating the need for additional treatment options, including multiple formulations. Although transdermal therapies have numerous advantages in the treatment of nervous system disorders, only one FDA-approved transdermal treatment (methylphenidate) for ADHD in children and adolescents is currently available.
